# Comparative Study of the Antioxidant Activity of the Conformers of *C*-tetra(4-methoxyphenyl)calix[4]resorcinarene

**DOI:** 10.3390/ijms251810010

**Published:** 2024-09-17

**Authors:** Laura Angélica Maldonado-Sanabria, Ivette Nicole Rodriguez-Saavedra, Ingrid Valentina Reyes-Peña, Alver Castillo-Aguirre, Mauricio Maldonado, Almudena Crespo, Miguel A. Esteso

**Affiliations:** 1 Departamento de Química, Facultad de Ciencias, Universidad Nacional de Colombia, Sede Bogotá, Carrera 30 No. 45-03, Bogotá 111311, Colombia; lmaldonados@unal.edu.co (L.A.M.-S.); ivrodriguezs@unal.edu.co (I.N.R.-S.); ireyes@unal.edu.co (I.V.R.-P.); aacastilloa@unal.edu.co (A.C.-A.); mmaldonadov@unal.edu.co (M.M.); 2 Universidad Católica de Ávila, Calle Los Canteros s/n, 05005 Ávila, Spain; almudena.crespo@ucavila.es; 3 U.D. Química Física, Universidad de Alcalá, 28805 Alcalá de Henares, Spain

**Keywords:** calix[4]resorcinarene, *crown* conformer, *chair* conformer, antioxidant activity

## Abstract

*C*-tetra(4-methoxyphenyl)calix[4]resorcinarene was synthesized by hydrochloric acid-catalysed cyclocondensation of resorcinol and 4-methoxybenzaldehyde. Under these conditions, the reaction produces a conformational mixture of *crown* and *chair* structural conformers, which were separated and characterized by chromatographic and spectroscopic techniques. The antioxidant activity of both conformers was measured by using the DPPH assay, through which it was observed that the *chair* conformer showed greater antioxidant activity (IC_50_ = 47.46 ppm) than the *crown* conformer (IC_50_ = 78.46 ppm). Additionally, it was observed that the mixture of both conformers presented lower antioxidant activity than either conformer in isolation. The results found suggest that the *chair* conformer has efficient antioxidant activity that makes it a potential target for further research.

## 1. Introduction

Oxidative stress involves an imbalance between natural antioxidant mechanisms and the production of reactive species derived from oxygen and nitrogen (RONS) [1]. RONS are free radicals with unpaired electrons, which are highly active in reacting chemically with other molecules. An excess of RONS can damage cellular lipids, proteins or DNA, inhibiting their normal function [2,3]. Consequently, oxidative stress is implicated in a variety of human diseases including cancer [4,5], neurodegenerative diseases [6,7,8], cardiovascular diseases [9,10] and many other pathologies [11]. Antioxidants are molecules that can neutralize free radicals by either accepting or donating electrons to eliminate the unpaired condition of the radical [12]. For example, many antioxidants have aromatic rings in their structure (such as polyhydroxy-phenolic compounds) and can act through two different mechanisms: donating a hydrogen–free radical (H) or donating an electron, becoming a new radical [13]. Generally, these new radicals are more stable and can be easily neutralized [14].

In this context, there are several reports on supramolecular platforms, such as calixarenes, used for the design of synthetic antioxidants [15]. These supramolecules are a family of macrocyclic compounds with a variable number of phenol units linked by methylene bridges in the *ortho* position [16].

Within the calixarene family, calix[4]resorcinarenes occupy an important place in different fields of study in pharmaceutical sciences due to their structural properties, which allow them to be suitably selective for molecules and ions and to be used in a wide range of applications [17,18,19]. In solution and in the solid state, calix[4]resorcinarenes present several types of conformations, such as *diamond*, *saddle*, *boat*, *chair* and *crown*, these last two conformations being the most stable. The ease with which they can be chemically modified, through the introduction of different groups on the resorcinol units, constitutes an important focus of attention in the synthesis of supramolecular systems [20,21]. Among these reactions, the functionalization ones on the lower rim are the most used in the synthesis of functionalized calix[4]resorcinarenes, since they lead to an interesting type of compound with different conformations in solution and in the solid state, which allow multiple applications [22,23].

In the case of calix[4]resorcinarenes, since they are polyhydroxy-supramolecules, they offer a highly efficient scaffold for the development of antioxidants that can be tailored by varying the substituent functional groups [15,24]. Different calix[4]resorcinarenes have been studied to determine their antioxidant activity: tetranitroxides calix[4]resorcinarenes [24,25], vanillin derivatives [25,26], C-methoxyphenylcalix[4]resorcinarene [26,27] and others [17,27,28]. In general, these papers report the results of antioxidant assays of the conformational mixture obtained from the synthesis; none of the publications reports the results for each of the conformers separately.

Continuing with our studies of calix[4]resorcinarenes and their usefulness in the field of pharmaceutical applications [29,30,31], in this paper, we show the results for each one of the conformers (*chair* and *crown*) of *C*-tetra(4-methoxyphenyl)calix[4]resorcinarene and the evaluation of its antioxidant activity through the DPPH assay both separately and as a mixture. The studies suggest that the *chair* conformer has efficient antioxidant activity, while both the mixture and the *crown* conformer present moderate activity.

## 2. Results and Discussion

### 2.1. Obtaining the Conformers of C-tetra(4-methoxyphenyl)calix[4]resorcinarene

As mentioned above, the synthesis of calix[4]resorcinarenes can lead to a large number of conformational isomers that can occur in the course of the reaction at varying concentrations. Taking this into account, for this study, we chose *C*-tetra(4-methoxyphenyl)calix[4]resorcinarene (**1**) in order to explore the antioxidant activity of the conformational mixture as well as each of the pure conformers. For this purpose, (**1**) was obtained according to the procedure described in the literature, through the acid-catalysed cyclocondensation of 4-anisaldehyde with resorcinol in ethyl alcohol under reflux condition [32].

**Scheme 1:**
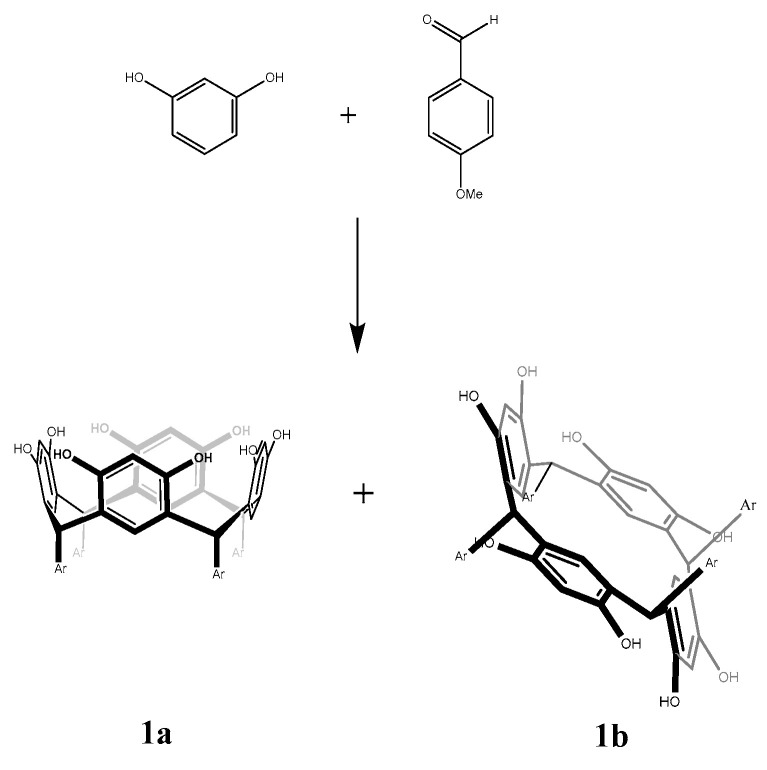
Synthesis of *C*-tetra(4-methoxyphenyl)calix[4]resorcinarene, including the *crown* (**1a**) and *chair* (**1b**) conformers.

**Figure 1:**
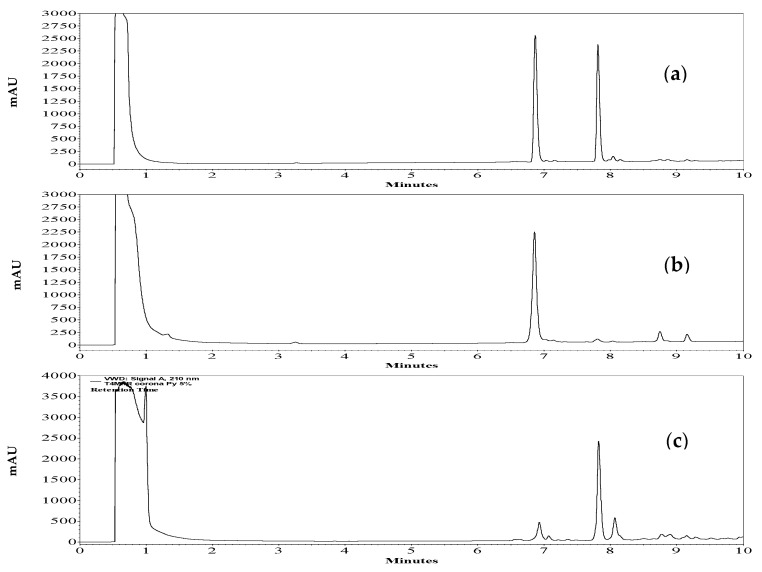
Chromatograms of the separation, via HPLC, of *C*-tetra(4-methoxyphenyl)calix[4]resorcinarene: (**a**) the conformational mixture, (**b**) the *chair* conformer and (**c**) the *crown* conformer.

In the reaction (Scheme 1), two main conformers were obtained. They were studied both in the conformational mixture and separately to determine their antioxidant capacity. The HPLC technique was used to analyse the reaction mixtures. The chromatographic method was carried out according to the conditions described in the experimental section. As shown in the chromatogram (Figure 1a), two peaks corresponding to the two conformers formed in the reaction were observed. These two conformers were efficiently separated, as can be seen in Figure 1b,c. Afterwards, they were purified following the method described in the experimental section.

Once the isomers were separated, the conformation of each product was established by spectroscopic analysis.

For the first product synthesized, **1a**, the FT-IR characterization showed the bands corresponding to the stretch of the O-H bond to 3390 cm^−1^, the C*_sp_*_2_-H bond > 3000 cm^−1^ typical of the aromatic ring and the C*_sp_*_3_-H bond < 3000 cm^−1^ of the hydrocarbon chain. Additionally, bands were observed at 1607 cm^−1^ (C=C aromatic) and 1115 cm^−1^ (C*_sp2_*-O).

The second product obtained in the synthesis, **1b**, exhibited in the FT-IR spectrum absorptions for the O-H stretch at 3360 cm^−1^, for the aromatic ring at 1604 cm^−1^ and for the C*_sp2_*-O stretching at 1115 cm^−1^.

**Figure 2:**
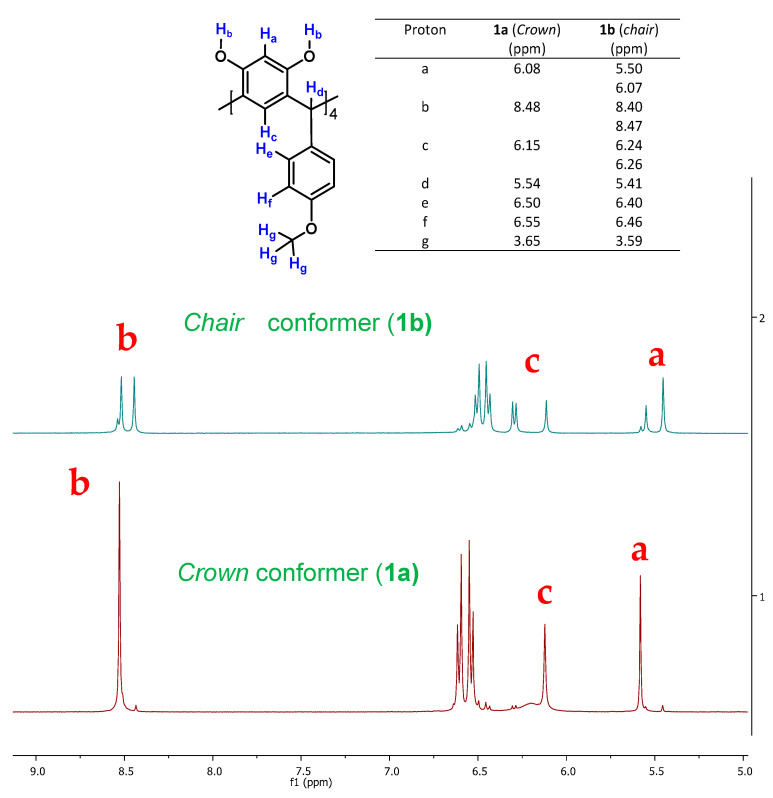
^1^H-NMR *C*-tetra(4-methoxyphenyl)calix[4]resorcinarene: *crown* conformer (**1a**) and *chair* conformer (**1b**). Signals a, b and c (in red) correspond to protons a, b and c of the resorcinol residue.

Furthermore, products **1a** and **1b** were characterized by ^1^H-NMR and ^13^C-NMR spectroscopy. The assignment of signals in the ^1^H-NMR spectrum for each conformer is summarized in Figure 2. Additionally, the number of signals observed in the ^13^C-NMR spectra for the carbons of each of the conformers, **1a** and **1b**, is consistent with the proposed structures. The synthesis and characterization of these isomers have been previously presented, and our spectroscopic data were in agreement with those reported [31].

In Figure 2, the signals of the ^1^H-NMR spectra obtained for each of the two conformers can be easily identified. As can be seen, the most affected signals are those of thea, b and c protons of the resorcinol residue. Thus, the highly symmetrical *crown* conformer (**1a**) shows singlet multiplicity signals for these protons at 6.08 ppm (proton a), 8.48 ppm (proton b) and 6.15 ppm (proton c), while the less symmetrical *chair* conformer (**1b**) presents two signals for each proton: 5.50 and 6.07 ppm for proton (a), 8.40 and 8.47 ppm for proton (b) and 6.24 and 6.26 for proton (c).

### 2.2. Antioxidant Activity

The antioxidant activity was determined for these *chair* and *crown* conformers, as well as for the conformational mixture of *C*-tetra(4-methoxyphenyl)calix[4]resorcinarene at a 1:1 ratio using the DPPH method. This test is based on the donation of electrons from antioxidants to neutralize the DPPH (1,1-diphenyl-2-picrylhydrazyl) radical. This is characterized by being a stable free radical due to the delocalization of an unpaired electron on the molecule. The delocalization gives rise to the appearance of an intense violet colour, with an absorption peak around 517 nm. When a DPPH solution is mixed with a substrate capable of donating a hydrogen atom, the DPPH radical is reduced, resulting in the loss of the violet colour [33,34].

**Figure 3:**
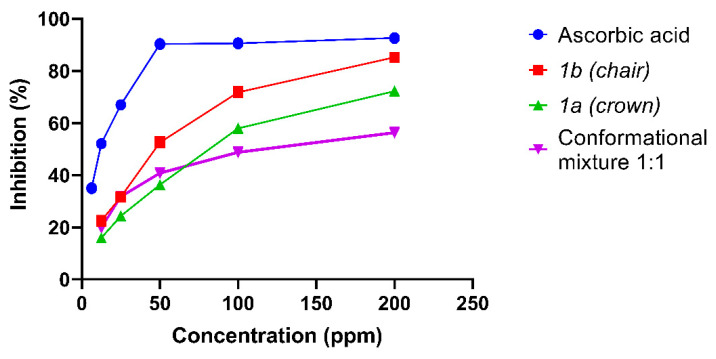
DPPH free radical inhibition for 1:1 conformer mixture, individual *C*-tetra(4-methoxyphenyl)calix[4]resorcinarene conformers (*chair* and *crown*) and positive control (ascorbic acid).

The inhibition percentage of DPPH free radicals versus concentration is illustrated in Figure 3 for all the compounds evaluated, with ascorbic acid serving as the positive control. As can be observed by analysing the graph, at low concentrations, there are no significant differences in the antioxidant activity of either the two conformers or the mixture of them. However, as the concentration increases, it can be observed that both isolated conformers show greater antioxidant efficacy than the conformational mixture (purple curve). Furthermore, the *chair* conformer (red curve) always exhibits greater antioxidant activity than the *crown* conformer (green curve). This difference in behaviour with concentration leads to the conclusion that the increase in solute–solute interactions, as a consequence of the increase in concentration, must favour the observed changes in the antioxidant activity.

Antioxidant strength is classified into four levels based on the IC_50_ values: very strong (IC_50_ < 50 ppm), strong (IC_50_: 50–100 ppm), moderate (IC_50_: 101–150 ppm) and weak (IC_50_: 250–500 ppm) [35]. Table 1 illustrates the IC_50_ values obtained, indicating that the *chair* conformer exhibits a very strong antioxidant effect with an IC_50_ of 47.46 ppm. It is closely followed by the *crown* conformer, which is classified as a strong antioxidant, with an IC_50_ of 78.46 ppm. However, the conformational mixture shows a lower antioxidant effect than expected based on the individual potential of each conformer, corresponding to a moderate level of antioxidant activity with an IC_50_ value of 121.48 ppm.

Considering this observation, we further analysed the type of interaction between the *chair* and *crown* conformers using Compusyn 8.0 software. This software is based on the median-effect equation and provides a quantitative definition through the combination index (CI) for the additive effect (CI = 1), synergy (CI < 1) and antagonism (CI > 1) of drug combinations [36].

**Figure 4:**
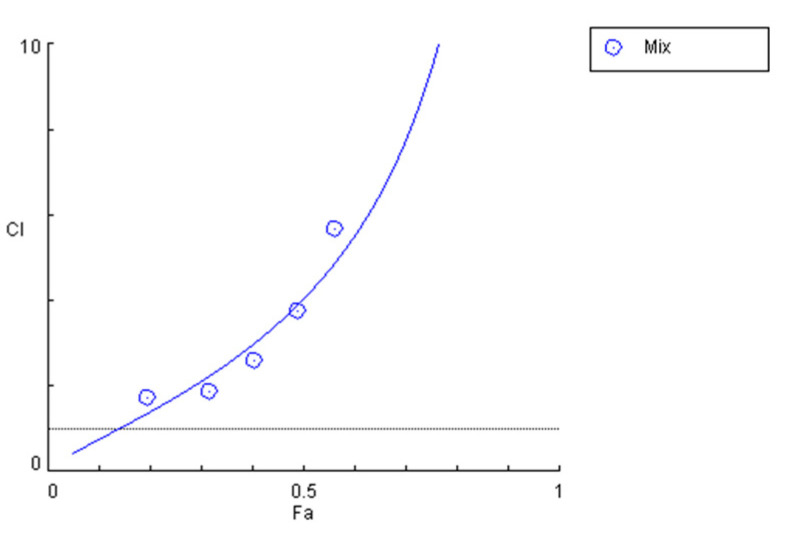
Fa–CI plot of the conformational mixture to evaluate antagonism. The combination index (CI) values are represented on the y-axis and the affected fraction (Fa) on the x-axis.

Compusyn 8.0 software calculated the combination index (CI) values for the experimental points assessed using the DPPH method for different conformational mixtures in a 1:1 ratio. As can be seen in Table 2, the CI values obtained confirm that these conformers exert an antagonistic interaction among themselves, with CI values greater than 1, in all the combinations evaluated. Furthermore, there is a noticeable trend of increasing CI values at higher concentrations, as observed in the fraction affected–combination index (Fa–CI) plot (Figure 4). The plot illustrates that as the fraction affected levels increase, the CI values also increase, indicating a strong antagonistic effect at higher concentrations.

**Figure 5:**
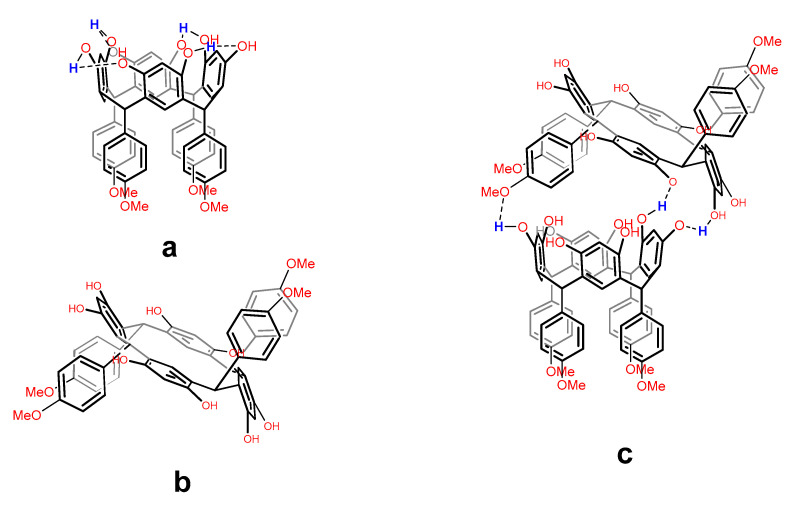
Hydrogen bonding interactions for **1a** (**a**), **1b** (**b**) and the conformational mixture (**c**).

In order to understand these results, it is necessary to take into account that phenols reduce oxidation rates through their hydroxyl groups. This is due to the transfer of a hydrogen atom to radical species, possibly by a concerted mechanism of hydrogen as a proton and one electron. However, these processes are significantly affected if phenols can generate hydrogen-bond-type interactions, either with solvents or with other types of solutes, which makes them practically inactive as antioxidants [13]. In this way, the low antioxidant activity of conformer **1a** can be explained, since the hydroxyl groups at the upper rim form stable hydrogen bonds that allow this compound to retain the crown conformation (Figure 5a), and the associated processes for antioxidant activity do not occur easily. In the case of conformer **1b**, it is observed that hydrogen bonds are not easily formed in solution; hence, the hydroxyl groups can present a proton transfer that favours their antioxidant activity (Figure 5b). Finally, in the case of the conformational mixture, it is possible that a strong interaction of the hydrogen-bond type occurs between **1a** and **1b**, which leads to the formation of molecular aggregates that significantly decrease the antioxidant activity (Figure 5c), thus generating an effect antagonistic to that which would be expected.

The results obtained here are consistent with observations previously made on similar molecules using density functional theory (DFT) and crystallographic data [37,38]. Thus, it has been observed that resorcinarenes such as those studied in this work tend mainly to adopt *chair* and *crown* conformations, which are the most stable isomers if the hydroxyl group is free. On the other hand, the substituent group on the methine carbon can significantly influence the antioxidant activity [39]; in our case, this group was the same (4-methoxyphenyl), which reinforces the concept that the conformation plays a determining role in the antioxidant activity of resorcinarenes.

## 3. Materials and Methods

### 3.1. Synthesis of C-tetra(4-methoxyphenyl)calix[4]resorcinarene

To a round-bottom flask, containing a 0.324 M solution of resorcinol in absolute ethanol, 1.10 mL of 4-anisaldehyde was added. Then, 3.4 mL of 37% HCl was added dropwise. The mixture was heated to reflux with constant stirring for 5 h. The reaction vessel was then placed in an ice bath to promote the formation of the precipitate of interest. It was then filtered under vacuum, and the solid obtained was washed with an ethanol–water mixture (1:1) until a neutral pH was reached. Finally, the solid compound was dried under reduced pressure with an Abderhalden drying pistol. The overall yield of the reaction was 98%.

The separation of the *crown* and *chair* conformers was carried out by using the crystallization technique. Briefly, 8 mL of DMSO was added to the solid obtained from the synthesis. This mixture was heated to reflux with constant stirring until its complete solubilisation; the heating was then stopped, and it was allowed to cool until it returned to room temperature and the crystals formed again. After this, vacuum filtration was carried out, washing the separated crystals with ethanol to remove all DMSO from them. Finally, to obtain the conformer solubilized in the liquid phase, cold vacuum filtration with ethanol was carried out. The conformers (*crown* and *chair*) of *C*-tetra(4-methoxyphenyl)calix[4]resorcinarene were characterized by ^1^H-NMR in DMSO-d6 at 400 MHz using a Bruker Avance 400 instrument.

*C*-tetra(4-methoxyphenyl)calix[4]resorcinarene (*crown*, **1a**) was obtained as a reddish solid, at a yield of 13.13%. Its spectroscopic properties were as follows. IR (ATR/cm^−1^): 3390 (OH), 2972 (C*_sp3_*-H), 1604 (C=C aromatic), 1115 (C*_sp2_*-O). ^1^H-NMR δ (ppm): 3.65 (s, 12 H, OCH3), 5.54 (s, 4H, ArCH), 6.08 (s, 4H, ArH *ortho* to OH), 6.15, (s, 4H, ArH *meta* to OH), 6.50 (d, 8H, *J* = 8 Hz, ArH), 6.55 (d, 8H, J = 8 Hz, ArH), 8.48 (s, 8OH, ArOH). ^13^C-NMR, δ (ppm): 54.8, 56.3, 102.2, 112.8, 120.9, 129.6, 129.8, 137.9, 152.7, 156.7.

*C*-tetra(4-methoxyphenyl)calix[4]resorcinarene (*chair*, **1b**) was obtained as a white solid, at a yield of 84.86%. Its spectroscopic properties were as follows. IR (ATR/cm^−1^): 3360 (OH), 2972 (C*_sp3_*-H), 1604 (C=C aromatic), 1115 (C*_sp2_*-O). ^1^H-NMR, δ (ppm): 3.59 (s, 12 H, OCH3), 5.41 (s, 4H, ArCH), 5.50 (s, 2H, ArH *ortho* to OH), 6.07 (s, 2H, ArH *ortho* to OH), 6.24 (s, 2H, ArH *meta* to OH), 6.26 (s, 2H, ArH *meta* to OH), 6.40 (d, 8H, *J* = 8 Hz, ArH), 6.46 (d, 8H, *J* = 8Hz, ArH), 8.40, (s, 4OH, ArOH), 8.47 (s, 4OH, ArOH). ^13^C-NMR, δ (ppm): 41.3, 54.6, 101.7, 112.5, 121.5, 129.9, 131.9, 136.5, 152.5, 156.6.

### 3.2. Chromatographic Analysis

RP-HPLC analyses were carried out on a Chromolith RP-18e (50 cm × 4.6 mm) using an Agilent 1200 liquid chromatograph (Agilent, Omaha, NE, USA). A gradient range of solvent B (0.05% TFA in acetonitrile) in solvent A (0.05% TFA in water) was used as follows: 20/20/100/100/20% B at 0/18/18/22/22, L/min. Detection was performed at 210 nm, and the flow rate was 2 mL/min.

### 3.3. DPPH Antioxidant Assay

1,1-Diphenyl-2-picryl-hydrazyl (DPPH) was obtained from Sigma-Aldrich (St. Louis, MO, USA). Its stability was verified by calibration as follows: a stock solution of 100 ppm was prepared in analytical grade methanol, and from it, DPPH solutions were prepared at concentrations of 10, 14, 18, 22, 26 and 30 ppm, which were analysed in an EMC-11-UV spectrophotometer at the maximum wavelength of 517 nm.

To perform the antioxidant activity assays, dilutions in DMF were prepared, at concentrations of 200, 100, 50, 25 and 12.5 ppm, of each of the two conformers (*crown* and *chair*) of *C*-tetra(4-methoxyphenyl)calix[4]resorcinarene, as well as of the mixture of both conformers. The test was carried out by adding 0.5 mL of the corresponding calix[4]resorcinarene solution and 2 mL of 20 ppm DPPH solution. The resulting mixture was incubated at 30 °C for 30 min with ultrasonication. Finally, absorbance measurements were taken in triplicate on a UV-Vis spectrophotometer, at a wavelength of 517 nm. The results were analysed using GraphPad Prism version 8.0.0 for Windows, and Compusyn 8.0 software was used to analyse the drug interactions [40].

## 4. Conclusions

In the present work, the synthesis and separation of the two conformers (*chair* and *crown*) of *C*-tetra(4-methoxyphenyl)calix[4]resorcinarene are shown. Once the two conformers were separated, they were characterized by HPLC and NMR techniques, which showed that both conformers had a high degree of purity.

On the other hand, the antioxidant activity of both conformers as well as that of the 1:1 conformational mixture was evaluated by using the DPPH protocol. The results showed that the *chair* conformation of *C*-tetra(4-methoxyphenyl)calix[4]resorcinarene possesses a higher free radical scavenging capacity (IC_50_ = 47.46 ppm) and shows greater antioxidant activity than the *crown* conformer (IC_50_ = 78.46 ppm). Finally, when the conformational mixture is used, the antioxidant activity decreases. A possible explanation for this behaviour is that hydrogen bond formation at the upper rim of the *crown* conformer prevents hydrogen–free radical (H) or electron donation, which significantly decreases antioxidant activity. This observation is consistent with the presented mechanisms of antioxidant activity for polyhydroxy-phenolic compounds.

## Figures and Tables

**Table 1:** IC_50_ values of the evaluated compounds.

Substance	IC_50_ (ppm)
Chair conformer	47.46 ± 0.45
Crown conformer	78.46 ± 0.35
Conformational mixture 1	121.48 ± 3.80
Ascorbic acid	13.03 ± 0.08

^1^ Mixture of *chair* and *crown* conformers (1:1).

**Table 2:** Combination index (CI) values for a 1:1 mixture of *chair* and *crown* conformers.

*Chair* Conformer (ppm)	*Crown* Conformer (ppm)	CI Value
12.5	12.5	1.73
25	25	1.86
50	50	2.58
100	100	3.76
200	200	5.69

## Data Availability

The data obtained is presented in the article.

## References

[1] Forman H.J., Zhang H. (2021). Targeting oxidative stress in disease: Promise and limitations of antioxidant therapy. Nat. Rev. Drug Discov..

[2] Valko M., Leibfritz D., Moncol J., Cronin M.T.D., Mazur M., Telser J. (2007). Free radicals and antioxidants in normal physiological functions and human disease. Int. J. Biochem. Cell Biol..

[3] Salisbury D., Bronas U. (2015). Reactive Oxygen and Nitrogen Species. Nurs. Res..

[4] Arfin S., Jha N.K., Jha S.K., Kesari K.K., Ruokolainen J., Roychoudhury S., Rathi B., Kumar D. (2021). Oxidative Stress in Cancer Cell Metabolism. Antioxidants.

[5] Reuter S., Gupta S.C., Chaturvedi M.M., Aggarwal B.B. (2010). Oxidative Stress, Inflammation, and Cancer: How Are They Linked?. Free Radic. Biol. Med..

[6] Chen X., Guo C., Kong J. (2012). Oxidative stress in neurodegenerative diseases. Neural Regen. Res..

[7] Kim G.H., Kim J.E., Rhie S.J., Yoon S. (2015). The Role of Oxidative Stress in Neurodegenerative Diseases. Exp. Neurobiol..

[8] Singh A., Kukreti R., Saso L., Kukreti S. (2019). Oxidative Stress: A Key Modulator in Neurodegenerative Diseases. Molecules.

[9] Dubois-Deruy E., Peugnet V., Turkieh A., Pinet F. (2020). Oxidative Stress in Cardiovascular Diseases. Antioxidants.

[10] Peoples J.N., Saraf A., Ghazal N., Pham T.T., Kwong J.Q. (2019). Mitochondrial Dysfunction and Oxidative Stress in Heart Disease. Exp. Mol. Med..

[11] Liguori I., Russo G., Curcio F., Bulli G., Aran L., Della-Morte D., Gargiulo G., Testa G., Cacciatore F., Bonaduce D. (2018). Oxidative Stress, Aging, and Diseases. Clin. Interv. Aging.

[12] Lü J.-M., Lin P.H., Yao Q., Chen C. (2009). Chemical and Molecular Mechanisms of Antioxidants: Experimental Approaches and Model Systems. J. Cell. Mol. Med..

[13] Foti M.C. (2007). Antioxidant Properties of Phenols. J. Pharm. Pharmacol..

[14] Ali Al-Mamary M., Moussa Z. (2021). Antioxidant Activity: The Presence and Impact of Hydroxyl Groups in Small Molecules of Natural and Synthetic Origin. Antioxidants-Benefits, Sources, Mechanisms of Action.

[15] Lee J.-S., Song I., Shinde P.B., Nimse S.B. (2020). Macrocycles and Supramolecules as Antioxidants: Excellent Scaffolds for Development of Potential Therapeutic Agents. Antioxidants.

[16] Español E.S., Villamil M.M. (2019). Calixarenes: Generalities and Their Role in Improving the Solubility, Biocompatibility, Stability, Bioavailability, Detection, and Transport of Biomolecules. Biomolecules.

[17] Jain V.K., Kanaiya P.H. (2011). Chemistry of calix[4]resorcinarenes. Russ. Chem. Rev..

[18] Castillo-Aguirre A., Esteso M.A., Maldonado M. (2020). Resorcin[4]arenes: Generalities and Their Role in the Modification and Detection of Amino Acids. Curr. Org. Chem..

[19] Tunstad L.M., Tucker J.A., Dalcanale E., Weiser J., Bryant J.A., Sherman J.C., Helgeson R.C., Knobler C.B., Cram D.J. (1989). Host-Guest Complexation. 48. Octol Building Blocks for Cavitands and Carcerands. J. Org. Chem..

[20] Iwanek W., Wzorek A. (2009). Introduction to the Chirality of Resorcinarenes. Mini-Rev. Org. Chem..

[21] Böhmer V., Kraft D., Tabatabai M. (1994). Inherently chiral calixarenes. J. Incl. Phenom. Mol. Recognit. Chem..

[22] Tero T.-R., Suhonen A., Salorinne K., Campos-Barbosa H., Nissinen M. (2013). The Missing Member of the Partially O-Alkylated Resorcinarene Family: Synthesis and Conformation of Methyl Tetramethoxy Resorcinarene. Org. Lett..

[23] Helttunen K., Shahgaldian P. (2010). Self-assembly of amphiphilic calixarenes and resorcinarenes in water. New J. Chem..

[24] Vovk A.I., Shivanyuk A.M., Bugas R.V., Muzychka O.V., Melnyk A.K. (2009). Antioxidant and Antiradical Activities of Resorcinarene Tetranitroxides. Bioorganic Med. Chem. Lett..

[25] Oliveira C., Meurer Y., Oliveira M., Medeiros W., Silva F., Brito A., Pontes D., Andrade-Neto V. (2014). Comparative Study on the Antioxidant and Anti-Toxoplasma Activities of Vanillin and Its Resorcinarene Derivative. Molecules.

[26] Budiana I., Gusti M., Ngurah B.I.G.M. (2018). Synthetic C-Methoxyphenyl Calix [4] Resorcinarene and Its Antioxidant Activity. J. Appl. Chem. Sci..

[27] Bishnoi A., Chawla H.M., Pant N., Mrig S., Kumar S. (2010). Evaluation of the Radical Scavenging Activity of Resorcinarenes by DPPH• Free Radical Assay. J. Chem. Res..

[28] Abosadiya H., Hasbullah S., Mackeen M., Low S., Ibrahim N., Koketsu M., Yamin B. (2013). Synthesis, Characterization, X-Ray Structure and Biological Activities of C-5-Bromo-2-Hydroxyphenylcalix[4]-2-Methyl Resorcinarene. Molecules.

[29] Pineda-Castañeda H.M., Maldonado-Villamil M., Parra-Giraldo C.M., Leal-Castro A.L., Fierro-Medina R., Rivera-Monroy Z.J., García-Castañeda J.E. (2023). Peptide-Resorcinarene conjugates obtained via Click Chemistry: Synthesis and antimicrobial activity. Antibiotics.

[30] Ramírez G., Cadavid-Montoya N.A., Maldonado M. (2023). Evaluation of a Resorcinarene-Based Sorbent as a Solid-Phase Extraction Material for the Enrichment of L-Carnitine from Aqueous Solutions. Processes.

[31] Matiz C., Castillo-Aguirre A., Maldonado M. (2024). Synthesis of C-tetra(aryl)resorcin[4]arenes using various types of catalysts under solvent free conditions: A comparative study. Green Chem. Lett. Rev..

[32] Castillo-Aguirre A., Pérez-Redondo A., Maldonado M. (2020). Influence of the hydrogen bond on the iteroselective O-alkylation of calix[4]resorcinarenes. J. Mol. Struct..

[33] Munteanu I.G., Apetrei C. (2021). Analytical Methods Used in Determining Antioxidant Activity: A Review. Int. J. Mol. Sci..

[34] Alam M.N., Bristi N.J., Rafiquzzaman M. (2013). Review on in vivo and in vitro methods evaluation of antioxidant activity. Saudi Pharm. J..

[35] Jumina J., Siswanta D., Zulkarnain A.K., Triono S., Priatmoko P., Yuanita E., Fatmasari N., Nursalim I. (2019). Development of C-Arylcalix[4]resorcinarenes and C-Arylcalix[4]pyrogallolarenes as Antioxidant and UV-B Protector. Indones. J. Chem..

[36] Zhang N., Fu J.N., Chou T.C. (2015). Synergistic combination of microtubule targeting anticancer fludelone with cytoprotective panaxytriol derived from panax ginseng against MX-1 cells in vitro: Experimental design and data analysis using the combination index method. Am. J. Cancer Res..

[37] Zhang Y., Kim C.D., Coppenes P. (2000). Does *C*-methylcalix[4]resorcinarene always adopt the crown shape conformation? A resorcinarene/bipyridine/decamethylruthenocene supramolecular clathrate with a novel framework structure. Chem. Commun..

[38] Shebitha A.A., Sreejith S.S., Sherly Mole P.B., Nithya Mohan N., Avudaiappan G., Hiba K., Priya K.S., Sreekumar K. (2020). Facile synthesis, X-ray diffraction studies, photophysical properties and DFT-D based conformational analysis of octa and dodecacyanomethoxycalix[4]resorcinarenes. J. Mol. Struct..

[39] Sathiyaseelan K., Muthu Prabhu A.A., Rajendiran N. (2024). Photophysical, Antioxidant, Antibacterial and NBO, LOL, ELF Analysis of Alkyl Groups Substituted Calix[4]resorcinarenes. J. Fluoresc..

[40] Chou T. (2006). Theoretical basis, experimental design, and computerized simulation of synergism and antagonism in drug combination studies. Pharmacol. Rev..

